# The protective effects of carboxyhemoglobin during the resuscitation from hemorrhagic shock in rats

**DOI:** 10.18632/oncotarget.18768

**Published:** 2017-06-28

**Authors:** Hongyu Liu, Songyan Yu, Yaojun Peng, Xin Chang, Xinguang Yu

**Affiliations:** ^1^ Department of Neurosurgery, Chinese PLA General Hospital, Beijing 100853, China; ^2^ Department of Endocrinology, Chinese PLA General Hospital, Beijing 100853, China; ^3^ Key Laboratory of Cancer Center, Chinese PLA General Hospital, Beijing 100853, China; ^4^ Department of Clinical Laboratory, Weihai Municipal Hospital, Weihai 264200, Shandong, China

**Keywords:** resuscitation from hemorrhagic shock, CO-red blood cells resuscitation, oxidative injury, inflammatory reaction

## Abstract

**Aim:**

This study was aimed to explore the effects of carboxyhemoglobin on reperfusion injury in hemorrhagic shock, as well as its action time and related mechanisms.

**Results:**

CO-RBC group showed milder oxidative injury than O2-RBC group. CO reperfusion did not show advantages in functions of kidney and lung during resuscitation. The level of *Bax* was decreased in CO-RBC group, especially in early CO-RBC group. Moreover, the autophay-related gene *Beclin-1* was down-regulated in CO-RBC and early CO-RBC groups. The inflammation was severer in CO-RBC resuscitation group.

**Materials and Methods:**

The hemorrhagic shock model rats were randomly divided into: the hemorrhagic shock group (*n* = 6); the O2-red blood cells (O_2_-RBC) group (*n* = 6), perfused with O_2_-RBC 1 h after ischemia; CO-RBC group (*n* = 12), perfused with CO-RBC 1 h after ischemia; and early CO-RBC group (*n* = 12), perfused with CO-RBC 30 min after ischemia. The reperfusion injuries were evaluated through anti-reactive oxygen species (ROS), inflammatory action, organ function, cell apoptosis and autophagy.

**Conclusions:**

Carboxyhemoglobin not only relieves the oxidative injury and inhibites apoptosis and autophagy, but also aggravates inflammatory reactions during reperfusion. The action time of carboxyhemoglobin may be an influencing factor for reperfusion outcomes.

## INTRODUCTION

Hemorrhagic shock is easily induced by bleeding from trauma, rupture of esophageal varices, peptic ulcer in clinic [1, 2]. Rapid blood supplement is the primary rescue measure, which can recover the microcirculatory perfusion and oxygen supply in a short time [3]. However, there will be uncontrolled systemic inflammatory reactions over blood transfusion, namely reperfusion injuries, which is characterized by a large number of oxygen free radicals, leading to organ damages and dysfunction [4, 5]. Therefore, it is important to avoid reperfusion injury in patients with hemorrhagic shock.

Reperfusion injury is a complex process with the involvement of oxidative stress, inflammatory response, cell apoptosis, autophagy, etc. However, the mechanisms underlying reperfusion injury remain unclear. Recently, accumulating studies are devoted to reduce reperfusion injury. The application of carbon monoxide (CO) is one of the hotspots. Evidence has shown that the oxygen supply recovery is not the earliest and the most critical demand, which could be delayed by carboxyhemoglobin (COHb) retransfusion during resuscitation from shock [6]. CO shows cytoprotective effects in hemorrhagic shock/resuscitation animal model via inhibiting inflammatory reactions [7]. In addition, heme oxygenase 1 (HO-1) is well known to play protective roles in inflammatory reactions and oxidative damage [8, 9]. CO, as a byproduct of HO-1 catalysis, can activate the soluble guanylate cyclase and transform GTP into cGMP at molecular level, playing important roles in relaxing smooth muscles and inhibiting platelet aggregation [10, 11]. Moreover, CO can also relieve the inflammatory reactions and suppress apoptosis via activating p38/MAPK signaling pathway [12]. In addition, it is reported that CO-RBC can improve the amelioration of hepatic ischemia-reperfusion injury induced by hemorrhage and resuscitation via hepatic CYP protection [13]. However, no studies have comprehensively explored the role of COHb on reperfusion injury and its related mechanisms.

This study was attempted to explore the protective function of CO in reperfusion injury, as well as its effects on oxidative damage, inflammatory reactions, organ function, cell apoptosis and autophagy using the hemorrhagic shock/resuscitation rat model. The resuscitation was implemented with infusion of autoblood mixed with CO. The current study might explore the role of CO in resuscitation from hemorrhagic shock and its related mechanisms which might provide a new insight into the management of reperfusion in hemorrhagic shock patients.

## RESULTS

### The decreased activities of RBC-SOD and lung-POD with COHb interference

**Figure 1:**
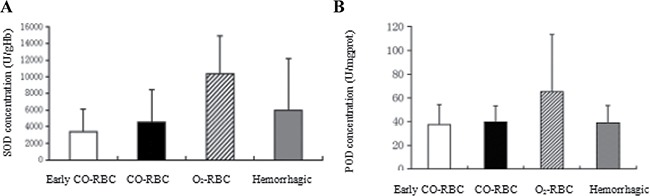
The effects of COHb on the antioxidative system during resuscitation from hemorrhagic shock The abdominal aortic blood was collected from the rats to detect the SOD activity. And a part of the right lung was selected to determine the POD activity. (**A**) SOD activity in RBC. (**B**) POD activity in lung tissues. Data were shown as mean ± SD.

Oxidative injury is a major portion of the ischemia reperfusion injury, which was assessed by two antioxidases SOD and POD in the study. The SOD activity was higher in O_2_-RBC resuscitation group (10341.16 ± 4576.96) than those in hemorrhagic shock group (5999.33 ± 6151.11) and CO-RBC resuscitation group (4555.44 ± 3880.48). In addition, the SOD activity in early CO-RBC resuscitation group (3359.28 ± 2768.96) was close to that in CO-RBC group (Figure 1A). The results of POD activity in all groups were similar to those of SOD (Figure 1B). COHb significantly decreased the activities of SOD and POD in the hemorrhagic shock group compared with O_2_-RBC resuscitation group, and early or delayed CO-RBC resuscitation showed no difference on antioxidation (Figure 1).

### The increased expression of *TNF-α*, *IL-1β*, and *IL-6* at mRNA level with COHb intervention

**Figure 2:**
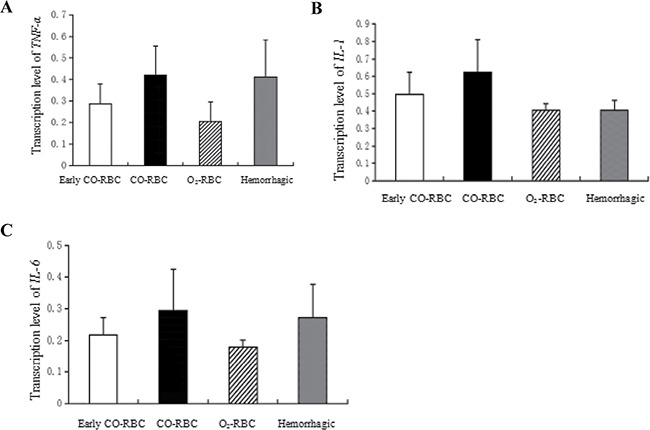
The effects of COHb on mRNA expression of *TNF-α*, *IL-1β*, and *IL-6* All these genes were extracted from the spleen and determined using RT-PCR. Data were presented as mean ± SD.

The study measured the mRNA expression of several pro-inflammatory factor *NF-α*, *IL-1β*, and *IL-6* in spleen to estimate the inflammatory reaction-caused tissue injury , which was also a portion of ischemia reperfusion injury. Generally speaking, the levels of pro-inflammatory factors in O_2_-RBC resuscitation group were lower than those in hemorrhagic shock group and CO-RBC resuscitation group, and the later two groups had close expression levels of all factors except that *IL-1β* expression was higher in CO-RBC blood group than hemorrhagic shock group (*P* < 0.05). Besides, the levels of pro-inflammatory factors were lower in early CO-RBC resuscitation group than delayed CO-RBC resuscitation group. In short, COHb increased the mRNA levels of pro-inflammatory factors in hemorrhagic shock group compared with O_2_-RBC group, and expression of these factors was lower in early CO-RBC group than delayed CO-RBC group (Figure 2).

### The effects of COHb on functions of kidney and lung in resuscitation from hemorrhagic shock

**Figure 3:**
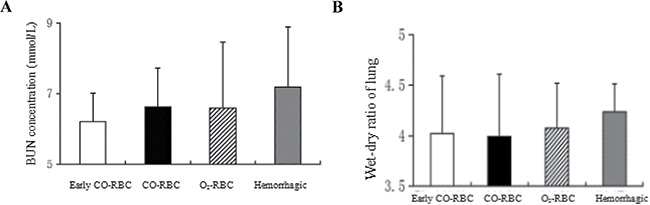
The influence of COHb on the functions and structures of kidney and lung The BUN concentration was determined with the abdominal aortic blood and the wet-dry ratio of lung was calculated with the left lung. (**A**) BUN concentration. (**B**) Wet-dry ratio of lung. All data were shown as mean ± SD.

Blood urea nitrogen (BUN) concentration a main indicator that could reflect the renal glomerulus filtration and other renal functions. In the study, BUN concentration was used to assess the influence of ischemia reperfusion on kidney function and determine the effects of COHb during the process. The BUN concentration in O_2_-RBC resuscitation group (6.60 ± 1.86) was lower than that in hemorrhagic shock group (7.19 ± 1.71), and close to that in CO-RBC resuscitation group (6.63 ± 1.10). Meanwhile, early CO-RBC resuscitation group (6.21 ± 0.81) showed low BUN concentration compared with CO-RBC resuscitation group (Figure 3A). COHb showed no influence on BUN concentration during resuscitation from hemorrhagic shock, however, the BUN concentration was lower in early CO-RBC resuscitation group rather than delayed CO-RBC resuscitation group.

Pulmonary edema is the main feature of acute lung injury induced by ischemia reperfusion. In the study, the effects of COHb on resuscitation from hemorrhagic shock was evaluated by the wet-dry ratio, an index of pulmonary edema. The lung wet-dry ratio was lower in O_2_-RBC resuscitation group (4.07 ± 0.45) than hemorrhagic shock group (4.24 ± 0.28), and close to CO-RBC resuscitation group (3.99 ± 0.62). In addition, similar lung wet-dry ratios were observed between early CO-RBC resuscitation group (4.02 ± 0.57) and CO-RBC resuscitation group (Figure 3B). COHb had no influence on pulmonary edema, and there was no difference in lung wet-dry ratio between early CO-RBC resuscitation group and delayed CO-RBC resuscitation group.

### Down-regulation of *Bax* mRNA and *Beclin-1* mRNA in lung tissues with COHb intervention

**Figure 4:**
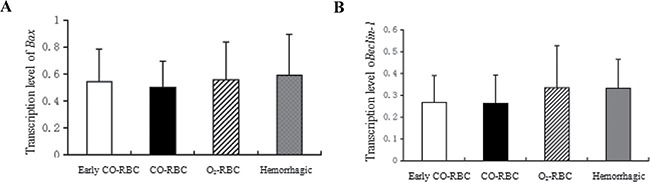
The effects of COHb on expression of *Bax* mRNA and *Beclin-1* mRNA *Bax* and *Beclin-1* were extracted from lung tissues and determined by RT-PCR using quantity one software. All data were presented as mean ± SD.

It is known that the majority of cells are apoptotic during the resuscitation from hemorrhagic shock. Thus, the present study determined the expression of *Bax* mRNA, a apoptosis-related gene, to investigate the effects of COHb on apoptosis in ischemia reperfusion injury. As shown in Figure 4A, the level of *Bax* mRNA was lower in O_2_-RBC resuscitation group (0.56 ± 0.28) than hemorrhagic shock group (0.59 ± 0.31), but higher than CO-RBC resuscitation group (0.50 ± 0.20). In addition, the *Bax* mRNA was significantly elevated in early CO-RBC resuscitation group compared with CO-RBC resuscitation group.

Autophagy represented a common stress response in cells. *Beclin-1*, an autophagy-related gene, was used to explore the effects of COHb on autophagy in ischemia reperfusion injury in the study. The expression of *Beclin-1* mRNA in O_2_-RBC resuscitation group (0.33 ± 0.19) was close to that in hemorrhagic shock group (0.33 ± 0.13), while the data was higher than that in CO-RBC resuscitation group (0.26 ± 0.13). Moreover, the *Beclin-1* mRNA level in early CO-RBC resuscitation group (0.27 ± 012) was close to that of CO-RBC resuscitation group (Figure 4B). The *Beclin-1* mRNA expression was significantly down-regulated by COHb in hemorrhagic shock group compared with blood resuscitation group, which had no difference between early CO-RBC resuscitation and delayed CO-RBC resuscitation.

## DISCUSSION

Oxidative damage is caused by a large number of oxygen free radicals which are generated with the recovery of oxygen supply after resuscitation from hemorrhagic shock, including lipid peroxidation of cell membrane, denaturation of proteins and nucleic acids [14]. Previous studies revealed that antioxidases had high affinity and reaction rate with oxygen free radicals [15]. In the current study, two antioxidases SOD and POD were selected as indicators to evaluate the oxidative damages reduced by resuscitation. The results showed that the activities of SOD and POD were both higher in O_2_-RBC resuscitation group. And the activity of SOD was lower in early CO-RBC and CO-RBC resuscitation groups. All of the data suggested that COHb could suppress the generation of oxygen free radicals through delaying the recovery of oxygen supply, thus reducing the oxidative injury. However, the specific damage should be assessed by further detection of malonaldehyde (MDA) level in tissues, which was also one of the limitations in the study.

Inflammatory reaction is considered as a pivotal reason for reperfusion injury. As a main organ of lymphocytes and monocytes, spleen can promote the production and release of inflammatory cytokines in early acute inflammatory reactions, thus the transcription of pro-inflammatory cytokines in the spleen may be applied to evaluate systemic inflammatory reactions [16, 17]. Previously researches indicated that CO could alleviate oxidative damage and inflammatory stress through activating p38 MAPK pathway [18, 19]. In our study, the transcription levels of pro-inflammatory cytokines were higher in CO-RBC resuscitation group than O_2_-RBC resuscitation group, but similar to hemorrhagic shock group, which was different from the previous studies. It is well known that CO is a toxic gas can not be ignored, and the affinity between CO and hemoglobin is 240 times higher than that of oxygen, and it can inhibit the release of oxygen. Based on the data, we speculated that CO caused tissue hypoxia, hypoxia induced necrosis, and then enhanced inflammatory response. However, further investigations were required to address the related issues.

Ischemia reperfusion injury can induce multiple organ dysfunction syndrome (MODS). The study investigated the role of COHb application in organ dysfunction during resuscitation from hemorrhagic shock via evaluating the structure and function of kidney and lung. The results showed that CO-RBC resuscitation group and O_2_-RBC resuscitation group had similar BUN concentration and lung wet-dry ratio, and both showed better structure and function of kidney and lung than hemorrhagic shock group. The renal compensation ability was strong and the BUN concentration would be increased massively only when the filtration rate of kidney glomerulus decreased to below 50% of normal values. Therefore, the ischemia reperfusion injury of kidney in our study might remain within its compensation ability (renal ischemia reperfusion). The present study did not find the protective effect of CO on lung, which might be related with the different ischemia reperfusion modes of between our study and previous studies.

Moreover, our study found that the transcription level of *Bax* in CO-RBC resuscitation group was lower than O_2_-RBC resuscitation group, which was accordant with the cell apoptosis detection conducted on these three groups after resuscitation by Pedro et al. [6]. And the results indicated that COHb could inhibit cell apoptosis, but the mechanisms were poorly known.

*Beclin-1* is a marker protein for autophagy in mammalians and participates in the formation of autophagosome membrane. Hence, the transcription level of *Beclin-1* is positively correlated with the induction level of autophagy. Studies have suggested that autophagy played a protective role in the ischemia reperfusion injury of the heart through cleaning the damaged mitochondria [20]. However, in the current study we found that the expression of autophagy-related gene was lower in CO-RBC group. Autophagy has dual characters. Some studies explained that autophagy could protect the cells during ischemia, but went against resuscitation during reperfusion, owing to the accumulation of mitochondria with structural and functional disorders in the damaged cells during ischemia, which might make autophagic responses more aggressive and lead to cell death during resuscitation [21, 22]. Thus, further investigations were still needed to explore the role of autophagy in resuscitation from hemorrhagic shock and the relationship between the expression of related-genes and CO reperfusion.

Whole blood resuscitation can recover the oxygen supply, which corrects the ischemia and hypoxia, but initiates oxidative injury. That is to say bodies presented comprehensive results of hypoxia injury and oxidative damage. The human COHb intervention can not only delay the recovery of oxygen supply and reduce oxidative damages but also recover blood supply and eventually accomplish reoxygenation. The appropriate action time of COHb is critical for the reperfusion outcomes. In this study, we found that the levels of cell apoptosis gene *Bax* were significantly lower in early CO-RBC group than that in the CO-RBC group, revealing that early application of CO reperfusion exhibited enhanced protective ability in cell apoptosis. Furthermore, we also found that the levels of inflammatory cytokines presented decreased trend in early CO-RBC group. The early application of CO might also suppress inflammatory reactions. Consequently, in order to improve the clinical outcomes of reperfusion among patients with hemorrhagic shock, further investigations were still needed to optimize the strategy for application of CO-RBC.

In conclusion, COHb plays important protective roles in oxidative injury and cell apoptosis through delaying the recovery of oxygen supply in ischemic tissues. However, COHb may aggravate inflammatory reactions. The outcomes of reperfusion may be influenced by the action time of COHb.

## MATERIALS AND METHODS

### Animals and reagents

Male SD rates (average body weight 293.61 g) were purchased from the Experimental Animal Center of the Academy of Military Medical Science. All animal studies were approved by the Institutional Care and Use Committee of the hospital.

The 10% chloral hydrate, 70 U/ml heparin sodium, recombinant human serum albumin (rHSA), total superoxide dismutase (T-SOD) Kit, peroxidase (POD) Kit, blood urea nitrogen (BUN) Kit and total protein Kit was purchased from Nanjing Jiancheng Bioengineering Institute. The Quantscript RT Kit and 2XTaq PCR Master Mix Kit were obtained from Tiangen Biotech (Beijing) Co., Ltd.

### Animal treatments and preparation of hemorrhagic shock model rats

A total of 36 adult male SD rats were randomly divided into 4 groups: hemorrhagic shock group (Hemorrhagic, *n* = 6), O_2_-red blood cells resuscitation group (O_2_-RBC, *n* = 6), CO-RBC resuscitation group (CO-RBC, *n* = 12) and early CO-RBC resuscitation group (early CO-RBC, *n* = 12). All animals were weighted and then anesthetized by 10% chloral hydrate (peritoneal injection, 0.3 ml/100 g). After that, the rats were fixed lying on the operation table. The right external jugular vein was selected from the median neck incision and injected with heparin sodium (70U/100 g), hemostasis by compression. The neck muscles were operated with blunt dissection to separate the left common carotid artery near the trachea. Blood withdrawal from the artery was complemented in 5min using a 10ml syringe and about 30% of the total blood volume (calculated as 6% of body weight) was collected. Then arterial occlusion was conducted.

### Preparation of COHb

The blood samples from donor rats were withdrawn into heparinized syringes (0.15 mL of 10,000 IU/mL heparin to 10 mL of blood) and centrifuged; it was then washed twice by resuspension in 5% rHSA and centrifugation (3000 g, 10 min). The formic acid and concentrated sulfuric acid (3:2) were mixed and then heated to prepare CO gas. When the CO concentration reached 1% (10000 ppm), mixed with balanced air (21% oxygen), and the concentration of CO was maintained at 250 ppm. The Hb of O_2_-RBC was adjusted to 8.6 g/dL. The CO-RBC was prepared using gentle CO bubbling for approximately 5 min. The levels of COHb in the blood were detected by OSM3 Hemoximeter (Radiometer Copenhagen, Copenhagen, Denmark).

### Resuscitative reperfusion

Half the draw-out blood (normal whole blood or CO-containing whole blood) was reperfused into the right external jugular vein. Rats in Hemorrhagic group were without any treatment; O_2_-RBC group rats were infused the normal whole blood into the right external jugular vein when the rats had ischemia 1 h. CO-RBC group were infused the CO-containing whole blood into the right external jugular vein when the rats had ischemia 1 h. Early CO-RBC group were infused the CO-containing whole blood when the rats had ischemia 30 min.

### Sample extraction of testing indexes

The spleen was resected from the median abdominal incision 1h after resuscitation (or 2 h after ischemia in the hemorrhagic shock group) and stored in liquid nitrogen for determining the mRNA expression of *TNF-α*, *IL-1β*, and *IL-6*. An aliquot of 2ml blood was withdrawn from the abdominal aorta to detect the BUN concentration and RBC-SOD activity. Both left and right lungs were excised by opening the thoracic cavity. Part of the right lung was stored in liquid nitrogen to examine the transcription of *Bax*, *Bcl-2*, *Beclin-1*, and the rest was used to detect the POD activity of lung tissues. The left lung was used to measure the wet-dry ratio of lung.

### Detection of antioxidase activity

Red blood cell extract was prepared by centrifugation of portal vein blood with 2 ml, then the erythrocyte SOD activity was detected by the nitrite formation method according to the instruction of total superoxide dismutase (T-SOD) Kit (Nanjing Jiancheng Bioengineering Institute).

After all the experiments, 1g lung tissue of rats were collected and treated with cold 5% CaCl_2_ solution, then put it in an ice water bath, the quality of the final concentration of 2%. Afterwards, centrifugation for 20 min in 4°C, 12 000 rpm, collecting supernatant, then using guaiacol colorimetric method to assay the activity of POD according to the instruction of peroxidase (POD) Kit (Nanjing Jiancheng Bioengineering Institute).

### Detection of blood urea nitrogen (BUN) concentration

After preparation of plasma samples, the BUN concentration was determined using the Fearon method.

### Wet-dry ratio of lung

The left lung was wiped by absorbent paper and weighed as wet weight. Then the lung was dried at 70°C for 12 h and weighted as dry weight. The wet-dry ratio could serve as an indicator for pulmonary edema.

### Quantitative real-time polymerase chain reaction (RT-PCR)

Total RNA was isolated from the rats’ spleens and lungs as previously described. The two-step PCR was performed. PCR conditions for *TNF-α*, *IL-1β*, and *IL-6* were as follows: pre-denaturation at 95°C for 30s, denaturation at 95°C for 5s, annealing at 60°C, and extension at 60°C for 34s, 30 cycles. And PCR for *Bax*, *Beclin-1* was taken under the following conditions: pre-denaturation at 94°C for 4 min, denaturation at 94°C for 30s, annealing at 55°C (*Bax*) or 51°C (*Beclin-1*), and extension for 30s, 30 cycles. *GAPDH* served as internal control, and the sequences of primers were summarized in Table 1.

**Table 1:** The primer sequences of related genes

Genes	Upstream primer (Sense)	Downstream primer (Antisense)
TNF-α	5′-AGGCTCCTCTCCGCCATCAAGA-3′	5′-TGGGCTCATACCAGGGCTT-3′
IL-1β	5′-GATGGCTGCACTATTCCTAATGC-3′	5′-AGACTGCCCATTCTCGACAAG-3′
IL-6	5′-TAGTCCTTCCTACCCCAACTTCC-3′	5′-TTGGTCCTTAGCCACTCCTTC-3′
Bax	5′-GGCGAATTGGAGATGAACTG-3′	5′-GTCACTGTCTGCCATGTGGG-3′
Beclin-1	5′-GGAGATGTTGGAGCAAATGAA-3′	5′-GTCGCATTGAAGACATTGGTT-3′
GAPDH	5′-CCACCACCATCTTCCAGGAG-3′	5′-CCTGCTTCACCACCTTCTTG-3′

### Statistical analysis

All data were presented as mean ± SD and analyzed using the Sigmaplot 12.5 software (Systat Software Inc., CA, USA). *P* < 0.05 was considered to be significant.
